# Genomic analyses of Northern snakehead (*Channa argus*) populations in North America

**DOI:** 10.7717/peerj.4581

**Published:** 2018-04-04

**Authors:** Carlee A. Resh, Matthew P. Galaska, Andrew R. Mahon

**Affiliations:** 1 Department of Biology, Central Michigan University, Mount Pleasant, MI, United States of America; 2 Department of Biological Sciences, Lehigh Univervsity, Bethlehem, PA, United States of America; 3 Department of Biology, Institute for Great Lakes Research, Central Michigan University, Mount Pleasant, MI, United States of America

**Keywords:** 2b-RAD sequencing, Aquatic invasive species, Population structure, Effective population size, Genomic analyses, Single-nucleotide polymorphism

## Abstract

**Background:**

The introduction of northern snakehead (*Channa argus*; Anabantiformes: Channidae) and their subsequent expansion is one of many problematic biological invasions in the United States. This harmful aquatic invasive species has become established in various parts of the eastern United States, including the Potomac River basin, and has recently become established in the Mississippi River basin in Arkansas. Effective management of *C. argus* and prevention of its further spread depends upon knowledge of current population structure in the United States.

**Methods:**

Novel methods for invasive species using whole genomic scans provide unprecedented levels of data, which are able to investigate fine scale differences between and within populations of organisms. In this study, we utilize 2b-RAD genomic sequencing to recover 1,007 single-nucleotide polymorphism (SNP) loci from genomic DNA extracted from 165 *C. argus* individuals: 147 individuals sampled along the East Coast of the United States and 18 individuals sampled throughout Arkansas.

**Results:**

Analysis of those SNP loci help to resolve existing population structure and recover five genetically distinct populations of *C. argus* in the United States. Additionally, information from the SNP loci enable us to begin to calculate the long-term effective population size ranges of this harmful aquatic invasive species. We estimate long-term *N_e_* to be 1,840,000–18,400,000 for the Upper Hudson River basin, 4,537,500–45,375,000 for the Lower Hudson River basin, 3,422,500–34,225,000 for the Potomac River basin, 2,715,000–7,150,000 for Philadelphia, and 2,580,000–25,800,000 for Arkansas populations.

**Discussion and Conclusions:**

This work provides evidence for the presence of more genetic populations than previously estimated and estimates population size, showing the invasive potential of *C. argus* in the United States. The valuable information gained from this study will allow effective management of the existing populations to avoid expansion and possibly enable future eradication efforts.

## Introduction

*Channa argus* (Anabantiformes: Channidae; Northern snakehead, Cantor 1842) is one of numerous fish species that have been introduced to the United States and is threatening native populations of organisms. This large, freshwater, piscivorous fish is native to China, Manchuria, southern Siberia, and Korea (Courtenay & Williams, 2004). They were imported to the US for the live food fish market and their introduction is mainly due to unauthorized intentional release of live individuals (Courtenay & Williams, 2004). Northern Snakehead were first reported in California in 1997, and the first established, reproducing population was documented in 2002 in a Maryland pond close to a Chesapeake bay tributary (Fuller & Benson, 2017). They spread rapidly due to their aggressive predation, rapid maturation rate, and ability to tolerate a wide range of environmental conditions (Orrell & Weigt, 2005; Wegleitner et al., 2016; Fuller & Benson, 2017). Since first detection approximately 20 years ago, *C. argus* populations have become established throughout the Potomac River, Chesapeake Bay, Hudson River and Delaware River basins (Fuller & Benson, 2017). Additionally, *C. argus* was discovered in Arkansas in April 2008 and persists following a failed eradication effort using rotenone (Fuller & Benson, 2017).

Biological invasions, such as the introduction and spread of northern snakehead in the United States, are occurring at an alarming rate and are a major contributor to loss of biodiversity, environmental change, and are economically expensive (Leung et al., 2002; Lodge et al., 2006). As the abundance of a non-indigenous species increases over time, eradication becomes increasingly more difficult and management becomes exceedingly more expensive. Successful removal of an invasive species is difficult, if not impossible, and expensive, especially after it becomes established (Lodge et al., 2006). Therefore, early detection and understanding of the population dynamics of the invasive species is important when only small, localized populations exist in a region (Lodge et al., 2006).

Genomic tools have become very important for monitoring and even early detection of rare species (Darling & Mahon, 2011). The data obtained through genomic methods can provide valuable information about the population structure of a species that is rare in a system and is especially advantageous because small sample sizes have been shown to be capable of determining fine scale population structure that accurately represent populations (Pujolar et al., 2013; Jeffries et al., 2016). Advances in high-throughput sequencing technology have decreased sequencing costs, but sequencing whole genomes can still be very expensive (Wang et al., 2012; Gautier et al., 2013; Andrews et al., 2016). Microsatellites have been a common tool for population studies because they are highly polymorphic, occur throughout the genome, and have a high mutation rate (Schlötterer, 2004; Wegleitner et al., 2016). However, microsatellite markers’ mutation pattern is complex, they can have high genotyping error rates, and while they occur throughout the genome, their density is low (DeFaveri et al., 2013). As a result, novel tools in the form of reduced representation genomic techniques have been developed that sample randomly, from throughout the genome (Davey et al., 2011). An example of this type of method involves restriction site associated DNA sequencing (RADseq). RADseq utilizes restriction enzyme anchored positions in the genome, allowing for the identification of thousands of single nucleotide polymorphism (SNP) loci (Davey & Blaxter, 2011). A specific type of RADseq, called 2b-RAD, developed by Wang et al. (2012), has made the process even more efficient by using type IIb restriction enzymes, such as *Bsa*XI and *Alf*I, which allows for custom reduction schemes to be tailored to the study in question. Thousands of SNP loci that are evenly distributed across the genome at high densities are recovered from 2b-RAD sequencing and provide robust results even when sample sizes are small (Pujolar et al., 2013; Nazareno et al., 2017). Jeffries et al. (2016) found that when compared to a microsatellite dataset, the RADseq approach produced more robust results and recovered finer population structure when they used a small sample size that included a large number of SNP loci. Additionally, Nazareno et al. (2017) were able to recover sufficient within-population genomic diversity using only 6–8 individuals, and only needed two individuals per population to be able to estimate population genetic structure, because they used a large number of polymorphic SNP loci. These and other studies (e.g., Willing, Dreyer & Van Oosterhout, 2012 and Senn et al., 2013) have shown that any limitations that may occur from small sample sizes can be overcome with an appropriate number of SNP loci. Restriction-site associated DNA digestion sequencing methods, such as 2b-RAD are inexpensive, rapid methods of analysis that generate a tremendous amount of data (e.g., hundreds to thousands of SNP loci from throughout the organism’s entire genome) that can be utilized to investigate population structure, estimate effective population size, estimate number of breeding individuals in a population, and to answer phylogeographic questions about populations (Pujolar et al., 2013; Pecoraro et al., 2016; Galaska et al., 2017a; Galaska et al., 2017b).

For *C. argus,* effective management and prevention of its further spread in North American waters depends upon knowledge of current population structure in the United States. Previous studies using microsatellite markers for this species have shown that a minimum of two genetically distinct populations are present in the eastern United States (Wegleitner et al., 2016). Using their microsatellite data, fish sampled from the Chesapeake Bay and Potomac River system comprise one population, whereas the fish present in the Hudson River basin comprise a genetically distinct population (Orrell & Weigt, 2005; Wegleitner et al., 2016). No Arkansas fish were included in previous population level studies. Therefore, the goal of this study was to determine the relationship of the Arkansas *C. argus* population to the eastern United States populations. Additionally, we chose to use a 2b-RAD sequencing method for genome scanning, to examine populations within the United States and these data will allow us to estimate the effective population size of established populations of *C. argus*’ sampled ranges. We hypothesize that fish in the established Arkansas region were introduced from existing populations in the eastern United States. Additionally, we anticipate the Arkansas population size of *C. argus* to be smaller than the population sizes in the eastern United States, due to an eradication attempt that occurred soon after their discovery. Resulting data will inform management agencies and aid in decisions regarding control of the populations, in order to avoid expansion and new introductions.

## Methods

### Sample collection and preparation

**Figure 1:**
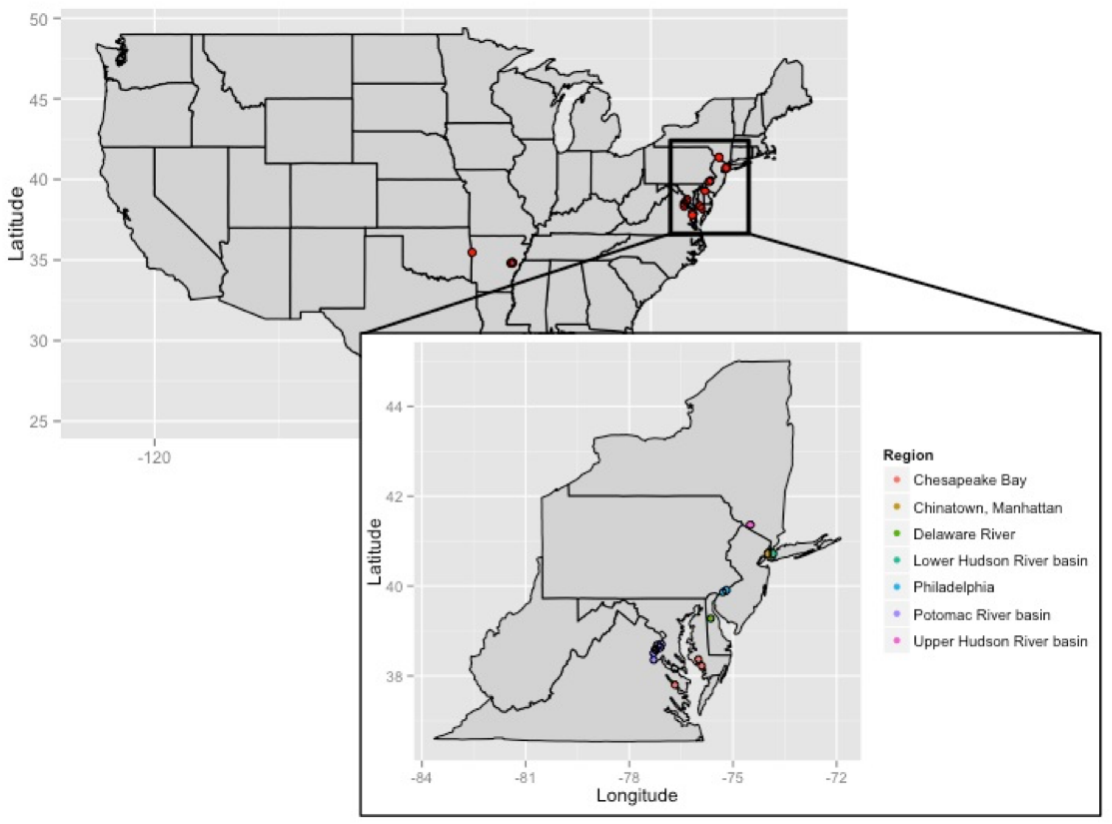
Collection locations for *Channa argus* individuals in the eastern United States and Arkansas.

Muscle tissue and fin clips (fresh, frozen, or preserved in 95% non-denatured ethanol) were collected from 165 *C. argus* from established populations in the United States by various individuals and management agencies (Fig. 1). This included collections from seven regions: Catlin Creek, part of the Upper Hudson River basin, Meadow and Willow Lakes in Queens, NY, part of the Lower Hudson River basin, fish from a market in Chinatown, in NYC, FDR park in Philadelphia, eight locations in the Potomac River, three rivers that are part of the Chesapeake Bay basin, and five locations in Arkansas (Table 1; see Table S1 for additional collection information). Genomic DNA was extracted from the muscle tissue and fin clips using the Qiagen DNeasy® Blood and Tissue kit (Qiagen, Valencia, CA, USA), following manufacturer’s protocols.

**Table 1:** Sample collection regions for *Channa argus*. *Channa argus* sampling regions. See Tables S1 and S2 for additional collection information.

**Location sampled**	**Number of individuals**
Upper Hudson River basin	59
Lower Hudson River basin	10
Chinatown, Manhattan	3
Philadelphia	21
Potomac river	50
Chesapeake bay	4
Arkansas	18

### Genomic data collection

Genomic DNA preparation followed the 2b-RAD protocol from Wang et al. (2012). Digestion of genomic DNA utilized the restriction enzyme *Alf*I. Two site-specific ligation adaptors (NC/NN) were used to employ a $ \frac{1}{4} $ reduction scheme. The adaptors bind to the two base-pair sticky end products of the *Alf*I restriction. The reduction scheme was chosen based on an approximate genome size of *C. argus* of 616–861 Mb to target approximately 2,500 SNP loci (A Libertini & F Krapp, 2017, unpublished data. Accessed from http://genomesize.com). Samples were dual barcoded with unique combinations before being sent for sequencing at HudsonAlpha Institute for Biotechnology Genome Services Laboratory (Huntsville, AL, USA) on an Illumina Hi-Seq 2500 using v4 chemistry, generating 50 bp single-end reads.

### Data analyses

Raw Illumina reads were demultiplexed by sample, quality filtered and aligned against a custom *de novo* reference sequence as outlined in Wang et al. (2012) using scripts from Dr. Eli Meyer (Oregon State University) (https://github.com/Eli-Meyer) and the software package Stacks v.1.35 (Catchen et al., 2011). The 2b-RAD data were filtered by loci to include samples with a minimum coverage of 20X. Homozygotic SNP loci were defined to have a maximum variance of 1% and heterozygotic loci had a minimum of 25% variance. Loci had to occur in 75% or more individuals within a sampling locality and be present in at least two localities to be processed. Loci not meeting these requirements were discarded prior to analyses.

Discriminant Analysis of Principal Components (DAPC) in the Adegenet v2.0.1 package (Jombart, 2008; Jombart, Ahmed & Bateman, 2011) in the R v3.4.1 statistical program (R Core Team, 2015) was used to analyze the SNP data to determine population structure of the putative *C. argus* populations. Adegenet conducts a series of Principal Component Analyses (PCA) on SNP data that is then retained to perform a Discriminant Analysis on all PCAs. Optimal clusters (*K*), likely representing populations, were identified through Bayesian information criterion (BIC) likelihood values from retained principal components. Visualization of the DAPC analyses was performed within the Adegenet package.

Population structure and admixture were assessed using the Landscape and Ecological Associations (LEA) v1.8.1 package in R (Frichot & François, 2015). *K* was estimated with the cross-entropy criterion and least squares estimates were used to calculate ancestry proportions (Frichot & François, 2015). Admixture was visualized by individual as bar charts and by locality as pie charts.

Further analyses involved generating summary statistics and analyzing genetic differentiation using the R package HIERFSTAT (ver. 0.04-22; Goudet, 2005), as well as calculating the effective population size (*N*_*e*_) of each of the *C. argus* populations. Initially, SNP data were analyzed assuming two populations (East Coast and Arkansas), based on the geographical distance between the eastern United States and Arkansas. Discrete populations were identified using both DAPC and LEA analyses. These analyses showed that the original seven sampling regions were not distinct genetic populations, but instead Chesapeake Bay and Potomac River were one genetic population (Potomac River basin population), and the Lower Hudson River basin and Chinatown fish were also one genetic population (Lower Hudson River basin population). Additional summary statistics were performed on each of the five putative populations and pairwise Fst differences were calculated to assess genetic differentiation between the newly identified populations. The effective population size of the East Coast and Arkansas populations were calculated using the following equation: *π* = 4∗*N*_*e*_∗*μ*, where *π* represents nucleotide diversity, *N*_*e*_ represents the effective population size, and *μ* represents the mutation rate of the SNPs (Tajima, 1983; Pujolar et al., 2013). Lastly, the effective number of breeders (*N*_*b*_) was calculated, using the following equation: *N*_*b*_ = *N*_*e*_(0.485 + 0.758∗log (adult lifespan∕age of first reproduction)) (Ruzzante et al., 2016).

## Results

A total of 23,695 single nucleotide polymorphism loci were recovered from 165 *C. argus*; quality filtering and SNP calling resulted in 1,007 independent SNP loci being retained in the final dataset.

**Figure 2:**
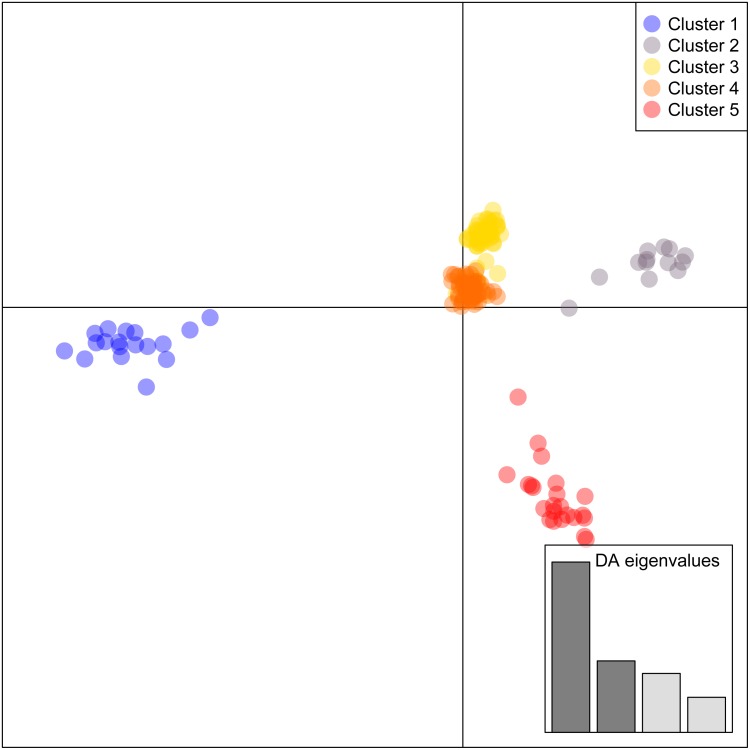
Discriminant Analysis of Principal Components for *Channa argus* single-nucleotide polymorphism data. Cluster 1 contains all 18 individuals from Arkansas. Cluster 2 contains 13 individuals: all 10 from Lower Hudson River basin and all three from the Chinatown, Manhattan fish market. Cluster 3 contains 52 individuals: 51 from Potomac River basin and one from Upper Hudson River basin. Cluster 4 contains 59 individuals: 57 from Upper Hudson River basin and two from Potomac River basin. Cluster 5 contains 23 individuals: one from Potomac River basin (Delaware River), one from Upper Hudson River basin, and all 21 individuals from Philadelphia.

Results of the DAPC analyses support five geographically and genomically distinct clusters or populations of *C. argus* in the United States (Fig. 2, Fig. S1). Cluster 1 contains 100% of the fish from Arkansas. Cluster 2 contains 100% of the fish from the Lower Hudson River basin and the Chinatown, Manhattan fish market. Cluster 3 contains 52 individuals, 96% of the fish collected from the Potomac River basin, and 1.7% of the fish collected from the Upper Hudson River basin. Cluster 4 contains 59 individuals, 96.6% of the fish from the Upper Hudson River basin, and 3.8% of the fish from the Potomac River basin. Cluster 5 contains 100% of the fish from Philadelphia, the only fish collected in the Delaware River, part of the Potomac River basin, and 1.7% of the fish from the Upper Hudson River basin.

**Figure 3:**
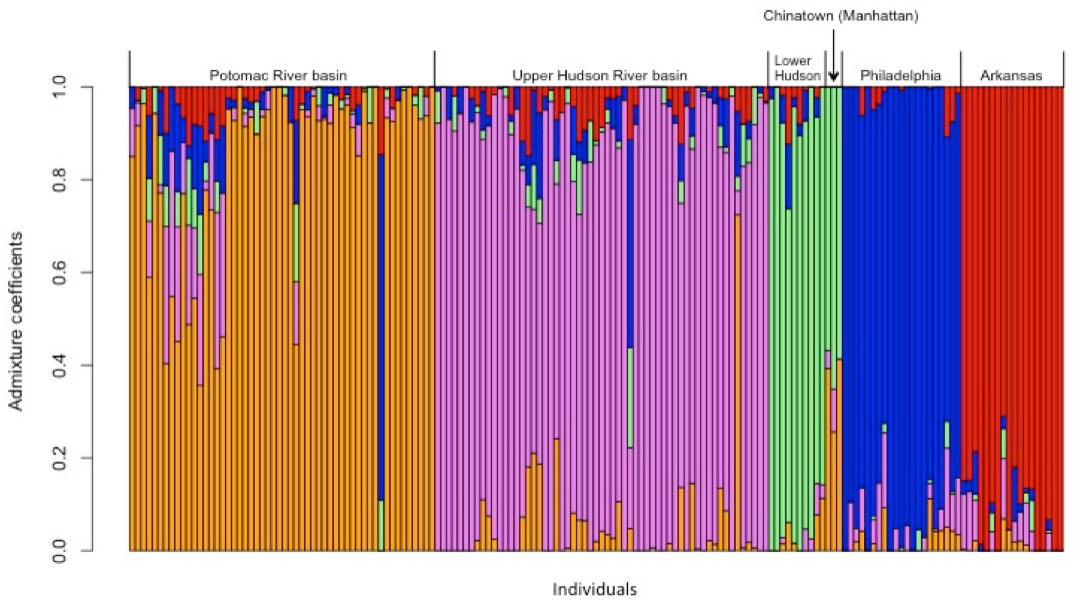
Admixture for *C. argus* populations (*K* = 5) in the United States. Each bar represents an individual (*X* axis) from each of the collection locations (top of figure). Individual admixture coefficients are represented in each column (*Y* axis).

**Figure 4:**
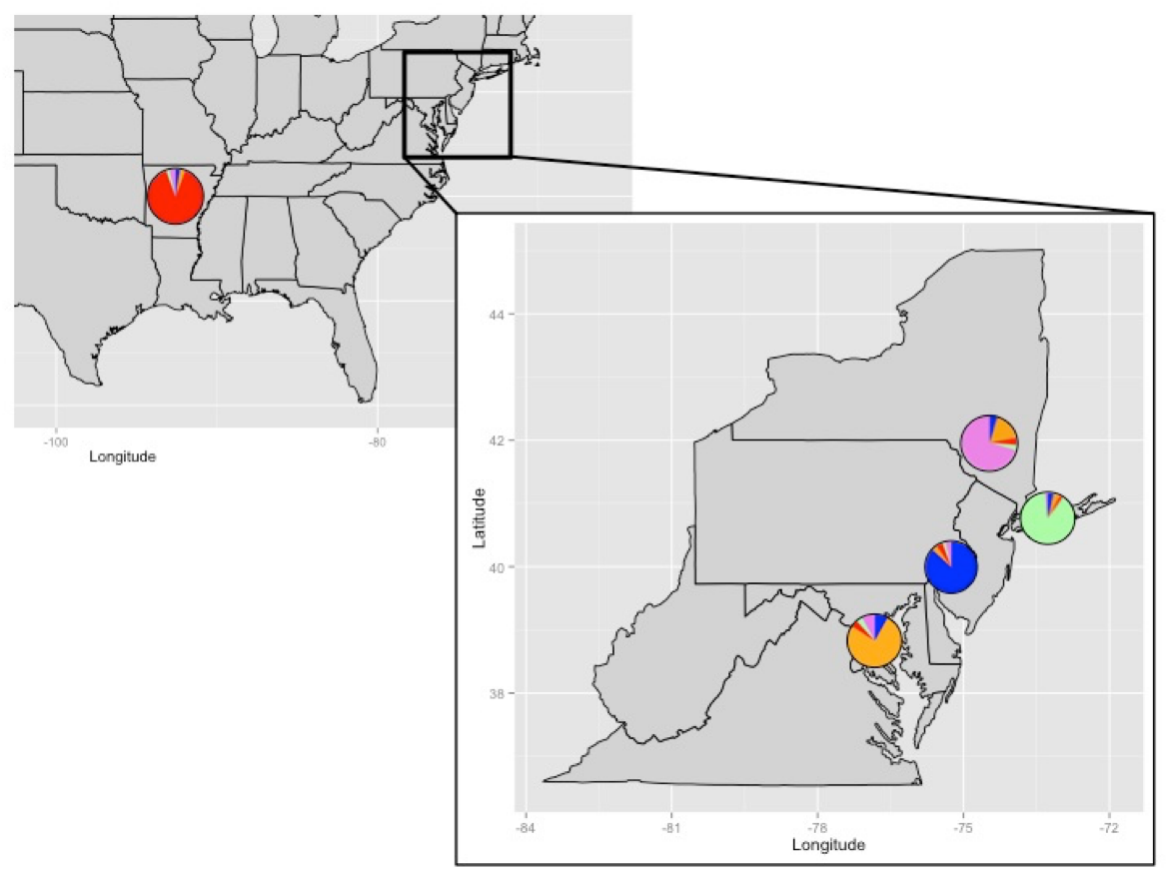
Admixture results by collection location. Pie charts represent the average admixture from each geographically distinct putative population (Arkansas, Potomac River Basin, Upper Hudson River Basin, Lower Hudson River basin, Philadelphia) as noted in the admixture plot of Fig. 3.

Additionally, the results of the LEA analyses also support the existence of five populations (*K* = 5, Fig. S2). Each of the populations is partially admixed, although the amount of gene flow varies among the populations (Figs. 3–4). The individuals from the fish market in Chinatown are statistically from the same population as most of the individuals in the Lower Hudson River basin, indicating they came from the same source (Fig. 3). One of the Upper Hudson River basin individuals and one of the Potomac River basin individuals share genotypes, determined statistically and shown in Fig. 3, with the Philadelphia fish. In contrast, the rest of the fish in the Potomac River basin are comprised primarily of a different ancestral genotype. The main ancestral genotype for all of the Arkansas fish is rare in the individuals from all of the East Coast populations.

Summary statistics of genetic diversity and genetic distances between the putative *C. argus* populations are given in Tables 2 and 3. The smallest genetic distance (0.08369683) is between the Upper Hudson River basin and the Philadelphia fish, whereas the largest genetic distance (0.18527692) is between the Potomac River and Arkansas populations. Heterozygosity is low for all putative populations. For the Potomac River basin population, the observed heterozygosity is lower than expected, whereas observed heterozygosity is the same as expected for the Upper Hudson River basin, Philadelphia, and Arkansas putative populations. Observed heterozygosity is higher than expected for only the Lower Hudson River basin putative population.

**Table 2:** Summary statistics for single nucleotide polymorphism loci. Individuals from Chinatown fish market excluded.

**Putative population**	*H*_*o*_	**Var**	**StdErr**	*H*_*e*_	**Var**	**StdErr**	*F*_*is*_	**Var**	**StdErr**
Potomac River basin	0.1167	0.0296	0.0078	0.1313	0.0326	0.0082	0.1429	21.3512	0.1226
Upper Hudson River basin	0.0662	0.0197	0.0069	0.0715	0.0187	0.0067	0.1185	25.9216	0.1698
Lower Hudson River basin	0.1643	0.0324	0.0059	0.1634	0.0252	0.0052	0.0266	15.0182	0.0329
Philadelphia	0.0862	0.0223	0.0049	0.0884	0.0223	0.0049	0.0094	32.6641	0.0524
Arkansas	0.0889	0.0301	0.0064	0.0893	0.0268	0.0060	0.0099	35.4852	0.0538

**Table 3:** Pairwise genetic distances between putative populations of *Channa argus*.

	**1**	**2**	**3**	**4**
**2**	0.17150707			
**3**	0.09671192	0.09378427		
**4**	0.16979238	0.08369683	0.09108509	
**5**	0.18527692	0.10232123	0.10871429	0.09207021

**Notes.**

1 Potomac River/Chesapeake Bay 2 Upper Hudson River basin 3 Lower Hudson River basin/Chinatown 4 Philadelphia 5 Arkansas

The long-term effective population sizes (*N*_*e*_) for each of the *C. argus* populations are shown in Table 4. The Chinatown fish market individuals are of questionable origin (i.e., not from a field site but from a captive market), thus they were excluded from this calculation. The Upper Hudson River basin *N*_*e*_ is the smallest, with an estimated range of 1,840,000–18,400,000 individuals (*π* = 0.0722 and the SNP mutation rate (*μ*) is 1 ×10^−8^–1 × 10^−9^ mutations per generation). The number of breeding individuals (*N*_*b*_) is estimated to be 2,287,120–22,871,200 individuals based on an adult lifespan of 10 years and age of first reproduction of one year (Odenkirk & Owens, 2007; Odenkirk et al., 2013). In contrast, the Lower Hudson River basin putative population has the largest *N*_*e*_, with an estimated range of 4,537,500–45,375,000 individuals (*π* = 0.1705), and an estimated range of 5,640,113–56,401,125 breeding individuals (*N*_*b*_). The *N*_*e*_ of Arkansas population is estimated to be 2,580,000–25,800,000 individuals (*π* = 0.0921) and *N*_*b*_ estimated range of 3,206,940–32,069,400 individuals.

**Table 4:** Nucleotide diversity and long term *N*_*e*_ and *N*_*b*_ values. Nucleotide diversity (*π*), long-term *N*_*e*_ and *N*_*b*_ estimates for each of the populations identified by DAPC. Chinatown samples have been excluded because they came from a fish market.

**Population**	*π*	*N*_*e*_	*N*_*b*_
Upper Hudson River basin	0.0736	1,840,000–18,400,000	2,287,120–22,871,200
Lower Hudson River basin	0.1815	4,537,500–45,375,000	5,640,113–56,401,125
Potomac River	0.1369	3,422,500–34,225,000	4,254,168–42,541,675
Philadelphia	0.1086	2,715,000–27,150,000	3,374,745–33,747,450
Arkansas	0.1032	2,580,000–25,800,000	3,206,940–32,069,400

## Discussion

The SNP data support the presence of five genetically distinct putative populations of *C. argus*: four in the eastern United States, and a distinct group in Arkansas. However, the Upper Hudson River basin population is not an active population. It was eradicated in 2008–2009 and considered successfully removed, based on subsequent monitoring by the New York State Department of Environmental Conservation. The tissues were collected as part of that effort and have been used here to attempt to determine if the Arkansas population originated from the Upper Hudson River basin. When compared to the East Coast snakehead populations that were sampled, the Arkansas population is distinct and mainly comprised of a unique ancestral genotype. Additionally, heterozygosity and the inbreeding coefficient is low for the Arkansas population, so there is little genetic variability, and inbreeding is occurring, showing that the population is geographically and genetically isolated from the east coast populations. This supports the possibility that the Arkansas population originated from a different source population than the source(s) of the eastern United States introductions. Thorough geographic sampling provides a clear distinction between the East Coast populations and the Arkansas population. The Arkansas population is the one that is the most genetically differentiated from the fish in the Potomac River and Lower Hudson River basins. It cannot be completely ruled out that the Arkansas population originated from an unidentified East Coast population not sampled in our study. Adding samples from *C. argus* native ranges could help to determine the source of the North American introductions, however, those samples were not available for the current study.

One of the principal goals of this study was to use a higher resolution method to answer the questions that remained unresolved from a previous study using microsatellites that sought to determine the number of genetic populations of *C. argus* in the eastern United States, as well as determine the source of the *C. argus* population in the Upper Hudson River basin. Wegleitner et al. (2016) suggested that two genetic populations exist in the eastern United States, and this was likely as a result of two separate introductions of *C. argus*. The results of this genomic scan study show a more complex story, providing evidence for the presence of five distinct populations of *C. argus*. Based on the shared ancestry and lack of genetic differentiation in the Potomac River basin population and fish from the Chinatown fish market, the fish in both geographic regions likely came from the same source. One individual sampled from the Potomac River basin shows strong genomic similarity to the *C. argus* in Philadelphia, as well as one individual from the Hudson River basin, suggesting introduction from one source, and subsequent population expansion. This is not surprising because of the close proximity of the Potomac River basin to Philadelphia (approx. 306 kilometers). However, because of the admixture that has occurred in each of the populations, it is not possible with the current dataset to determine how many introductions occurred.

## Conclusions

An accurate assessment of current population size is crucial for conservation efforts and management, along with making accurate models of population growth. This is the first estimate of long-term effective population size for the Arkansas and East Coast populations using SNP data and these estimates are based off the estimation method used by Pujolar et al. (2013) that calculates *N*_*e*_ based on nucleotide diversity in a population. Since they were first reported in Arkansas in 2008, it’s possible that individuals from the original source population could skew the *N*_*e*_ and *N*_*b*_ estimates if any of the fish used in this study are from the original introduction event. *Channa argus* has a relatively long lifespan and high fecundity in both native and introduced ranges. Previous studies have used *C. argus* individuals from the Potomac River basin that were estimated to be as old as 10.1 years (Odenkirk & Owens, 2007) and have been documented to have a maximum lifespan of 15 years in their native environment (Jiao et al., 2009). Adult female fecundity estimates are 22,000–51,000 in their native range (Amur River basin; Nikol’skiy, 1956) to 28,600–115,000 in an introduced population (Syr Dar’ya basin, Turkmenistan/Uzbekistan; Dukravets & Machulin, 1978; Fuller & Benson, 2017). Furthermore, they may spawn up to five times a year in their native range, while in an introduced population in Kazakhstan, they spawned 1–3 times per year (Courtenay & Williams, 2004). In addition to producing moderately sized egg clutches, *C. argus* guard their nests and young for up to nine weeks (Ling, 1977; Landis, Lapointe & Angermeier, 2011). Landis & Lapointe (2010) observed both male and female *C. argus* guarding eggs and fry for at least four weeks in a Potomac River tributary, including protecting them when they left the nest. Based on their SNP data, Pujolar et al. (2013) also estimated much higher values for long-term effective population size of the European eel than had previously been reported using microsatellite data. Common programs that provide *N*_*e*_ estimates using microsatellite data make assumptions that oversimplify microsatellite mutation patterns, which could result in low *N*_*e*_ values. Unfortunately, there are no other *N*_*e*_ estimates for *C. argus* populations in North America using microsatellites, nor does census information exist. Therefore, it is difficult to assess the accuracy of our *N*_*e*_ estimates. However, given the properties of SNP markers, the time since established in the United States (at least 15 years), the high genetic diversity in these populations, high reproductive potential, and extensive protection of offspring, it is not surprising that the long term effective population size estimates are so high.

According to the US Army Corps of Engineers (Grippo et al., 2017), *C. argus* has a low overall risk rating for the probability of establishment in the Great Lakes basin because they are currently located in Arkansas in low numbers and known to only occur in one river system. These results show that there are likely more fish in Arkansas than previously known, they may occur in more than one river system, and likely have become established. Despite a decrease in population size following an eradication attempt by the Arkansas Fish and Game Commission in 2009, the Arkansas *C. argus* effective population size is not the smallest of the five populations. *Channa argus* is an extremely environmentally tolerant species, and therefore was able to rebound from the eradication attempt, and currently has an effective population size within the range of the other populations, demonstrating its potential to quickly increase in abundance. Furthermore, Kramer et al. (2017) used global species distribution models and information about local habitats to assess the suitability of the Great Lakes for *C. argus* establishment and determined that the climate conditions throughout most of the Great Lakes would be suitable for their establishment. These results show that *C. argus* has established large effective populations along the East Coast of the United States and in Arkansas. Other aquatic invasive species with similar life history traits and habitat preferences, such as grass carp, quickly expanded their ranges after being introduced in Arkansas, spreading into the Mississippi River basin, and the Great Lakes basin within 50 years of their introduction. These results illustrate the potential for *C. argus* to quickly expand its range, like the grass carp and its relatives. Expansion could have major economic and environmental impacts on commercial and recreational fisheries, and native fish populations (Courtenay & Williams, 2004; Grippo et al., 2017).

The ability to develop effective management strategies to avoid further *C. argus* expansion and subsequent harm to the Mississippi River and Great Lakes basin ecosystems is enhanced by the results of this study. Source population information would aid in the development of a complete picture and the best resulting management strategies. That information in currently not available, but important conclusions and management recommendations can still be made based on our current dataset. These data provide evidence of gene flow between the Potomac River basin and Philadelphia populations. Previous studies have shown that this will make eradication efforts difficult due to the increased likelihood of recolonization (Abdelkrim, Pascal & Smadi, 2017; Farrington et al., 2017). However, the *C. argus* population in Arkansas is isolated from the East Coast populations, indicating recent establishment and little to no gene flow between Arkansas and the East Coast. Additionally, the main ancestral genotype present in the Arkansas population is rare in the East Coast populations, providing evidence for a different source population. If, as our data suggest, the Arkansas fish originated from a different source than the East Coast populations, then it is important to monitor and prevent possible means of introduction into Arkansas. Therefore, management efforts should focus on the entire population in Arkansas and should be handled differently than the East Coast populations. The isolation of the Arkansas population increases the likelihood of successful eradication, which is crucial to avoid expansion and subsequent harm to the Great Lakes basin.

##  Supplemental Information

10.7717/peerj.4581/supp-1 Fig. S1 Optimal K graph based on Bayesian information criterion (BIC) values for population estimation for discriminant analysis of principal components

10.7717/peerj.4581/supp-2 Fig. S2 Optimal K graph based on minimal cross entropy for admixture analyes performed in LEA (ver. 1.8.1; Frichot & François, 2015)

10.7717/peerj.4581/supp-3 Table S3 Collection information for samples utilized in the current study

## Additional Information and Declarations

### DNA Deposition

The following information was supplied regarding the deposition of DNA sequences: SRA sequence data are available in Genbank at the following accession numbers: SAMN08331753–SAMN08331787.
