# Supplementary figures and images for: Genomic analyses of Northern snakehead (Channa argus) populations in North America
Source: PeerJ. 2018 Apr 4;6:e4581. doi: 10.7717/peerj.4581 (PMC5889702; doi:10.7717/peerj.4581)

**Value of BIC  
versus number of clusters**

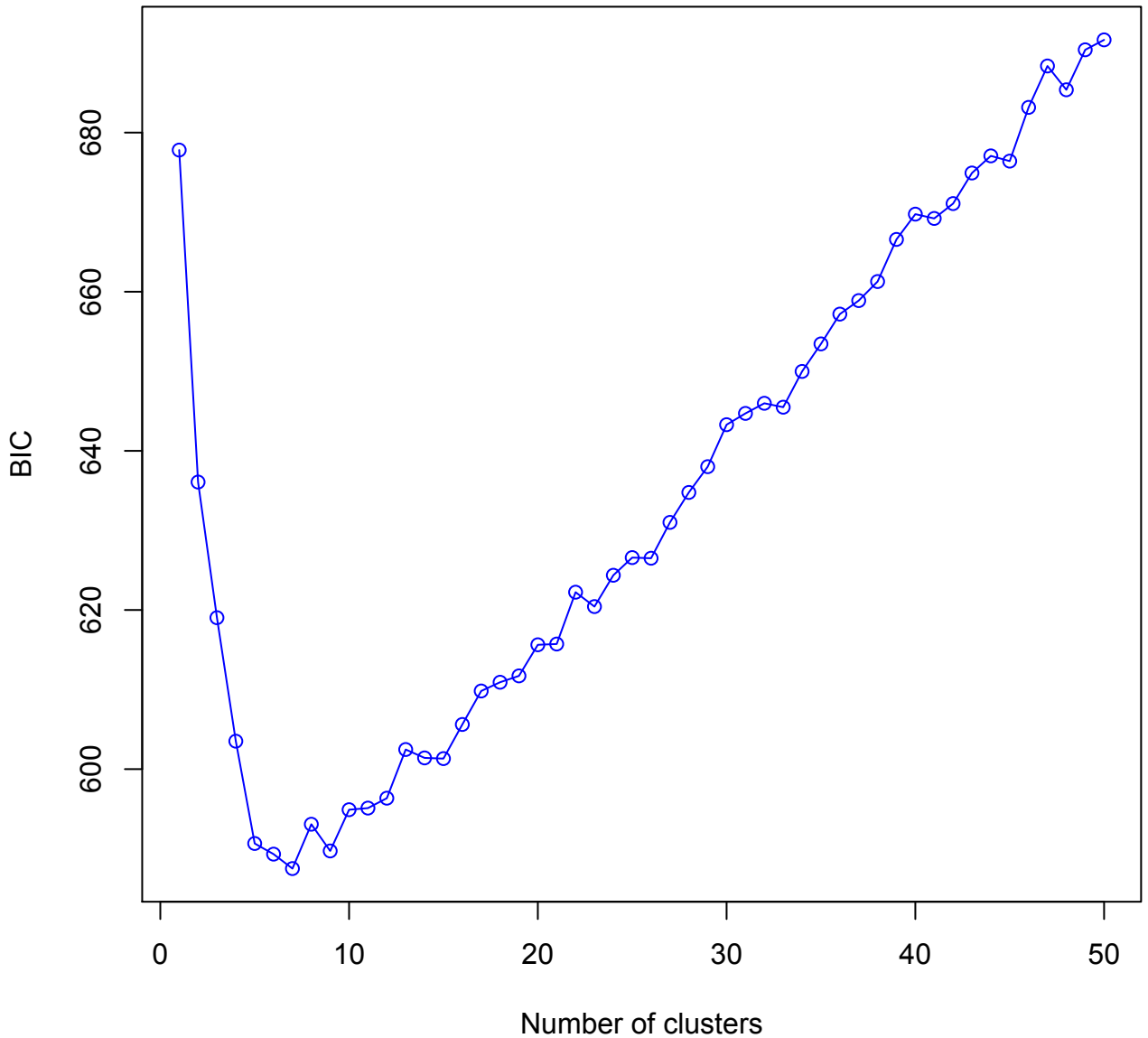

Supplement: Fig. S1 [file peerj-06-4581-s001.pdf]

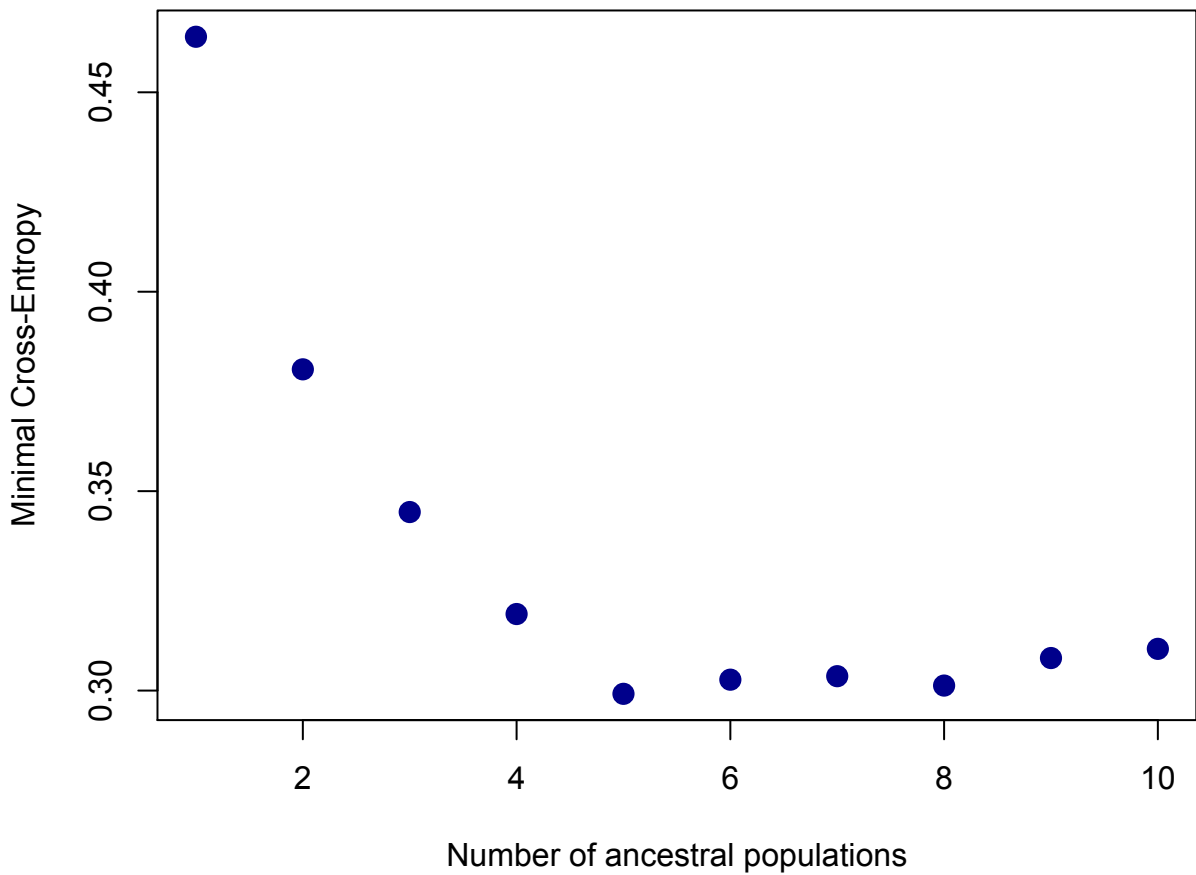

Supplement: Fig. S2 [file peerj-06-4581-s002.pdf]
